# Land vs. water HIIE effects on muscle oxygenation and physiological parameter responses in postmenopausal women

**DOI:** 10.1038/s41598-020-70599-6

**Published:** 2020-08-13

**Authors:** Kuei-Yu Chien, Nai-Wen Kan, Yi-Hung Liao, Wen-Ting Yang, Yong Yang

**Affiliations:** 1 Graduate Institute of Sports Science, National Taiwan Sport University, 250, Wenhua 1st Rd., Guishan District, Taoyuan City, 333325 Taiwan; 2 Center for General Education, Taipei Medical University, Taipei, Taiwan; 3 Department of Exercise and Health Science, National Taipei University of Nursing and Health Sciences, Taipei, Taiwan; 4 Graduate Institute of Athletics and Coaching Science, National Taiwan Sport University, Taoyuan, Taiwan

**Keywords:** Cardiovascular biology, Circulation, Respiration

## Abstract

Muscle oxygenation (MO) status is the dynamic balance between O_2_ utilization and O_2_ delivery. Low-impact high-intensity interval exercise MO responses in the exercise and recovery stage are still unclear. We compared the differences in MO and physiological parameters between high-intensity interval water-based exercise (WHIIE) and high-intensity interval land bike ergonomic exercise (LBEHIIE) in postmenopausal women. Eleven postmenopausal women completed WHIIE or LBEHIIE in counter-balanced order. Eight sets were performed and each exercise set included high intensity with 80% heart rate reserve (HRR) in 30 s and dynamic recovery with 50% HRR in 90 s. Muscle tissue oxygen saturation index (TSI), total hemoglobin (tHb), oxy-hemoglobin (O_2_Hb), and deoxy-hemoglobin (HHb) were recorded. Blood lactate, heart rate and rating of perceived exertion (RPE) were measured at pre and post-exercise. Under similar exercise intensity, RPE in WHIIE was lower than that in LBEHIIE. The heart rate in WHIIE was lower than that in LBEHIIE at 1 and 2 min post-exercise. During the dynamic recovery, TSI, tHb, and O2Hb in water were higher than on land. A negative correlation was found between the change in TSI and lactate concentration (r = − 0.664). WHIIE produced greater muscle oxygenation during dynamic recovery. Muscle TSI% was inversely related to blood lactate concentration during exercise in water.

## Introduction

Women become estrogen deficient in the post-menopausal period. This increases the risk for cardiovascular disease^1,2^. Numerous studies demonstrated that higher than moderate intensity exercise can reduce cardiovascular disease and the overall risk of death^3,4^. However, lack of time is one of the most important obstacles to regular exercise^5^. High-intensity interval training (HIIT) can achieve cardiovascular stimulation intensity within a short time period. HIIT is an exercise pattern that alternates high-intensity exercise periods with low-intensity recovery periods. High intensity is defined as above 80% HRmax^6^, and it is generally more common to use intensity greater than 80–95% HRmax^7–10^. Dynamic exercise intensity recovery is the moderate intensity of 50–60% HRR or of 60–70% VO_2_peak^11^.

Previous studies demonstrated that HIIT on land can effectively reduce post-menopausal women’s abdominal fat^12^, inflammatory markers^10^, insulin sensitivity, oxidative stress and cardiovascular disease risk^13^. Although there are many benefits to on-land HIIT, participants must endure higher joint impact and the risk for acute muscle soreness or muscle damage with weight-bearing exercise on land^14,15^. Furthermore, a decline in muscle power and muscle strength^16^ and increase in deleterious musculoskeletal conditions^17^ occurs after middle-age. The high-impact jumping exercise on land is too burdensome for postmenopausal women. The buoyancy of water can reduce the joint load and joint impact caused by exercise. Exercising in shallow water it will increase the resistance in the lower limbs, so that the lower limb workload increases. Bicycling is a probable exercise mode for increasing lower limb muscle strength for special groups with bone and joint dysfunction. The stationary bike is an easy, suitable exercise mode for the middle-aged and elderly perform HIIT^18^. Therefore, using water-based exercise or bike ergometer on land as high-intensity interval training modes for post-menopausal women is acceptable.

Previous study showed that water interval training enhances flexibility and anaerobic power while decreasing musculoskeletal impact placed on the ligaments, joints, and tendons^19^. The muscle damage from resistance exercise is also lower in water than on land^20^. Therefore, water-based exercises are suitable for special groups such as postmenopausal women and older adults^21^. Several previous studies showed that aquatic exercise training had cardiovascular benefits^22,23^, especially for those with high risk musculoskeletal conditions^24^.

Muscle oxygenation (MO) reflects the dynamic balance between oxygenation consumption and oxygen supply^25^. Study has shown that changing trends in muscle oxygenation and blood lactate concentration during incremental exercise loads agree with aerobic and anaerobic metabolism theories^26^. The greater the decrease in muscle oxygen saturation, the greater the amount of oxygen required for metabolism during exercise^25^. Study has demonstrated that the tissue saturation index (TSI) in muscle tissue gradually decreases with increasing exercise intensity^27^. Decreased TSI in muscle tissue leads to an increase in blood lactate concentration^26,28^. Lactate is an intermediate of glucose metabolism and its increase may be related to conscious fatigue^29,30^. Previous study indicated that water cycling exercises result in lower blood lactate accumulation or faster lactate reduction than do land cycling exercises^31^. The increase in water hydrostatic pressure induces heart blood volume during diastole greater than that on land, therefore the stroke volume is increased, and the blood flow to the main action muscle group is increased^32,33^. Study has also indicated that delta oxy-hemoglobin (O2Hb) strongly corresponds with local perfusion, and the total oxygen index corresponds with both local perfusion and deoxygenation^34^. Therefore, the lower lactate concentration in water exercises may be related to changes in the related MO parameters caused by the water’s physical factors such as buoyancy, hydrostatic pressure and so on.

As we know, there is no relevant study that compares the acute MO responses and physiological parameters with these two intermittent exercise modes in post-menopausal women. In the absence of such information, there is a lack of scientific evidence as a decision-making reference for a post-menopausal women curriculum designed for exercise instructors. The purpose of the present study compares the differences in MO and physiological parameters between similar high-intensity interval water-based exercises (WHIIE) and high-intensity interval land bike ergonomic exercise (LBEHIIE) in post-menopausal women. Moreover, the muscle oxygen saturation responses of water-based HIIT in the exercise and recovery stage are still unclear. There are no studies that report on the correlation between TSI and lactate concentration in water-based and land based bike ergonomic exercises. Therefore, this study also investigates the correlation between muscle TSI and blood lactate concentration. The hypothesis of this study was that heart rate, rating of perceived exertion (RPE), blood lactate concentration in WHIIE were lower than those from LBEHIIE. The MO in WHIIE was higher than those in LBEHIIE.

## Results

Table 1 shows no difference between WHIIE and LBEHIIE for HRR in main exercise and the dynamic recovery. The WHIIE heart rate reserve percentage was significantly lower when compared to those performed by LBEHIIE at the 1st minute and the 2nd minute post-exercise. The WHIIE RPE was significantly lower than that from LBEHIIE in the main exercise, immediately after exercise, and the 10 min post exercise.

**Table 1:** The heart rate and RPE responses following high-intensity interval exercise.

	WHIIE	LBEHIIE	*p*
**Pre exercise**			
HR	78.0 (74.7, 83.8)*	68.0 (66.3, 74.3)	0.003
RPE	8.0 (7.2, 9.0)	7.0 (7.0, 8.1)	0.084
**Main exercise**			
HRR (%)	73.5 (68.8, 77.2)	71.4 (68.7, 76.5)	0.594
RPE	12.4 (11.1, 13.3)*	13.9 (12.6, 14.7)	0.011
**Dynamic recovery**			
HRR (%)	45.9 (44.2, 48.3)	45.1 (42.2, 48.7)	0.182
RPE	11.0 (10.1, 12.1)	11.8 (10.5, 12.8)	0.327
**Post exercise**			
HRR (%)	74.0 (67.1, 82.2)	80.0 (68.0, 86.7)	0.207
RPE	12.0 (11.2, 14.1)*	14.0 (12.1, 15.3)	0.025
**Post 1 min exercise**			
HRR (%)	35.0 (28.6, 42.7)*	49.0 (43.2, 63.6)	0.006
RPE	11.0 (10.2, 12.7)	13.0 (10.5, 14.0)	0.215
**Post 2 min exercise**			
HRR (%)	25.0 (18.4, 33.4)*	37.0 (33.0, 51.0)	0.005
RPE	10.0 (9.5, 11.4)	11.0 (9.2, 12.4)	0.670
**Post 10 min exercise**			
HRR (%)	28.0 (20.2, 32.0)	32.0 (25.2, 43.1)	0.103
RPE	9.0 (7.7, 9.1)*	10.0 (8.2, 11.2)	0.028

*HR*, heart rate; *BPM*, beats per minutes; *RPE*, rating of perceived exertion; *WHIIE*, water high-intensity interval exercise; *LBEHIIE*, land bike ergonomic high-intensity interval exercise; *HRR*, heart rate reserve.

*Significantly from LBEHIIE (*p* < 0.05).

There was no difference in muscle oxygenation parameters between the water and land environments during the main exercise stage. The TSI, tHb (total hemoglobin) and O2Hb (oxy-hemoglobin) of the dynamic recovery stage in water-based exercise were significantly higher than that on land ergometer exercise (Table 2). The effect size and power values during dynamic recovery were respectively TSI: 0.55, 0.79; tHb: 0.53, 0.76; O_2_Hb: 0.63, 0.87.

**Table 2:** Muscle oxygenation related parameters in various exercise stage.

	WHIIE	LBEHIIE	*p*
**Pre exercise**			
TSI (%)	73.8 (70.4, 75.2)	74.1 (70.5, 77.1)	0.424
tHb (µM)	16.3 (9.9, 22.3)	8.7 (3.6, 22.8)	0.182
O_2_Hb (µM)	7.6 (3.4, 10.6)	4.2 (0.4, 12.9)	0.286
HHb (µM)	8.0 (5.8, 12.3)	2.4 (2.5, 10.6)	0.131
**Main exercise**			
TSI (%)	72.1 (67.6, 78.3)	71.9 (66.3, 81.5)	0.722
tHb (µM)	10.5 (3.0, 18.9)	6.1 (1.9, 15.0)	0.155
O_2_Hb (µM)	3.1 (− 0.5, 8.9)	2.1 (0.9, 5.1)	0.999
HHb (µM)	6.3 (2.2, 11.3)	2.7 (0.4, 10.4)	0.328
**Dynamic recovery**			
TSI (%)	79.0 (77.8, 81.6)*	78.0 (73.3, 80.1)	0.010
tHb (µM)	18.5 (12.1, 26.5)*	9.7 (5.9, 19.5)	0.013
O_2_Hb (µM)	14.9 (9.6, 18.6)*	9.1 (4.7, 11.7)	0.003
HHb (µM)	4.2 (2.4, 8.0)	1.4 (0.7, 8.4)	0.594
**Post exercise**			
TSI (%)	73.7 (69.3, 76.7)	69.8 (65.8, 79.0)	0.594
tHb (µM)	14.1 (8.8, 24.8)	11.8 (5.4, 27.7)	0.722
O_2_Hb (µM)	7.8 (5.8, 14.1)	3.6 (2.3, 14.1)	0.248
HHb (µM)	5.0 (2.7, 11.1)	4.8 (2.1, 14.6)	0.213
**Post exercise 5 min**			
TSI (%)	82.3 (80.2, 85.5)	80.4 (77.3, 83.6)	0.169
tHb (µM)	20.4 (14.1, 27.6)	21.3 (11.8, 28.7)	0.756
O_2_Hb (µM)	18.7 (13.7, 22.0)	17.4 (10.8, 21.2)	0.266
HHb (µM)	1.7 (0.1, 6.0)	3.0 (0.6, 7.9)	0.423

*WHIIE*, water high-intensity interval exercise; *LBEHIIE*, land bikeergonomic high-intensity interval exercise; *TSI*, tissue saturation index; *tHb*, total hemoglobin; *O*_*2*_*Hb*, oxy-hemoglobin; *HHb*, deoxy-hemoglobin.

*Significantly from LBEHIIE (*p* < 0.05).

The lactate concentration showed a lower trend in WHIIE than in LBEHIIE immediately and 10 min after exercise (*p* = 0.062). In the WHIIE condition the lactate concentration at 10 min after exercise increased by 146.7% (84.2, 232.0) compared with the lactate concentration before exercise, which was a trend of lower than the 277.8% (168.5, 463.7) of LBEHIIE (*p* = 0.062) (Table 3). The effect size were 0.40 and the power of lactate data were 0.55 in immediately and 10 min post-exercise.

**Table 3:** The lactate concentration responses in various stages.

	WHIIE	LBEHIIE	*p*
Pre exercise (mmol/L)	1.7 (1.4, 1.8)	1.3 (1.3, 1.6)	0.180
Post exercise (mmol/L)	5.7 (4.8, 6.2)	6.9 (5.3, 7.8)	0.062
Post 10 min exercise (mmol/L)	3.9 (2.7, 5.3)	5.7 (3.9, 7.5)	0.062
CPEI (%)	235.7 (189.1, 327.8)	338.5 (265.1, 453.6)	0.110
CP10E (%)	146.7 (84.2, 232.0)	277.8 (168.5, 463.7)	0.062

*WHIIE*, water high-intensity interval exercise; *LBEHIIE*, land bike ergonomic high-intensity interval exercise; *CPEI*, the change of post exercise, (Post exercise − Pre exercise)/Pre exercise*100; *CP10E*, the change in post 10 min exercise, (Post 10 min exercise − Pre exercise)/Pre exercise*100.

*Significantly from LBEHIIE (p < 0.05).

**Figure 1:**
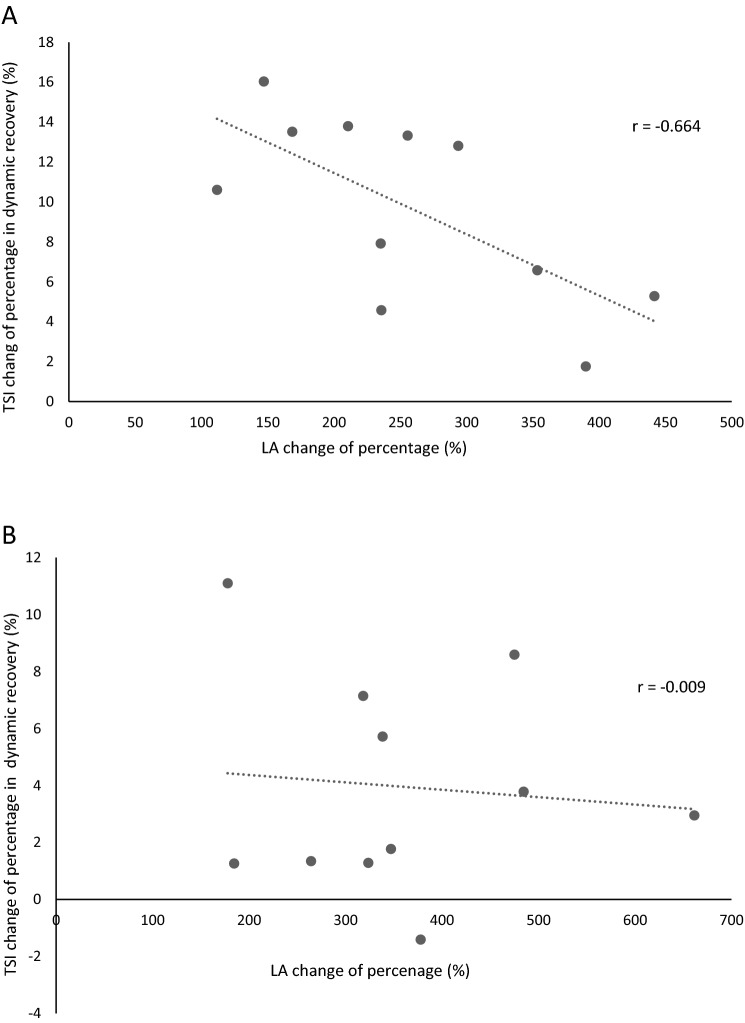
The correlation between the percentage changes in TSI and lactate concentration during dynamic recovery. (**A**) Water high-intensity interval exercise. (**B**) Land high-intensity interval exercise.

Figure 1A indicates that the TSI percentage change in the dynamic recovery stage at the WHIIE test was inversely related to the percentage increase in lactate concentration after exercise (r = − 0.664, p = 0.026). However, the amount of percentage change in TSI during the dynamic recovery stage in the LBEHIIE test was independent of the percentage increase in lactate concentration (r = − 0.009, p = 0.979) after exercise (Fig. 1B).

## Discussion

The main finding was that the RPE in WHIIE was lower than in LBEHIIE with similar exercise intensity during the exercise and at the 10th minute after exercise. A faster heart rate recovery rate occurred in WHIIE than in LBEHIIE. The muscle tissue oxygen saturation, tHb and O_2_Hb of the dynamic recovery stage in WHIIE were higher than in LBEHIIE. The more TSI increased during the dynamic recovery stage in WHIIE, the lower the lactate concentration rate increased after exercise.

In this study the WHITE heart rate was 73.5% HRR (68.8, 77.2), and LBEHIIE was 71.4% HRR (68.7, 76.5). The exercise intensity in both environments was in the high-intensity exercise target zone (60–89% HRR)^35^. The heart rate reserved percentages showed no significant difference between the water-based exercise and land ergometer exercise ether in the main exercise or in the dynamic recovery stage. That indicated that the intensities of the two environmental exercises were similar. It is worth noting that the heart rate reserve percentage of WHIIE was significantly lower than that of LBEHIIE at the 1st and 2nd minutes after exercise. A previous study demonstrated that short-term heart rate recovery after exercise was associated with parasympathetic regulation^36^. If there is more parasympathetic activity during recovery after exercise, the body can return to stability from the high heart rate, high blood pressure, and a large amount of muscle blood supply after strenuous exercise. That would be helpful to recovery after exercise.

RPE is a scale for the participant to engage in conscious exercise effort or fatigue sensation. The results from this study showed that the RPE of WHIIE was significantly lower than for LBEHIIE during the main exercise stage. This indicated that under similar exercise intensity, WHIIE had a lower self-conscious fatigue response than that of LBEHIIE. This phenomenon may be caused by the water buoyancy combined with the movement characteristics in the water. The greater the number of body parts immersed in the water, the more the buoyancy increases. This study designed a high-intensity intermittent jumping action exercise for the water. The water buoyancy assists the force required to jump upwards, thus making it easier for participants. In addition, due to the water buoyancy and drag, the quadriceps and hamstring muscles are activated, assisting with action execution when performing lower limb movements in the water. A previous study indicated that biceps femoral muscle activity in the water is higher than that on land. This activity increased with increasing water depth^37^. Conversely, when participants performed a land-based ergometer exercise, the quadriceps muscles are the major muscle group, so participants may be more likely to feel fatigued. At the end of the exercise and 10th minute after exercise, the WHIIE RPE values were still significantly lower than LBEHIIE. However, there was no significant difference in RPE between the 1st and 2nd minute after exercise. The significant difference RPEs during the 10 min period after exercise may result from the immediate WHIIE RPE being lower than LBEHIIE at the time of exercise. In addition, the heat transfer coefficient is 0.56 (J/s m °C) in water and 0.023 (J/s m °C) on the air. The water heat transfer coefficient is about 24 times that of air. In this study we wiped the surface moisture from the participants' body with a towel and did not change clothes after WHIIE. There was no difference in heart rate after ten minutes of exercise between WHIIE and LBEHIIE. The reason WHIIE showed lower RPE is that the residual moisture on the clothes increased the heat dissipation from the body surface, which makes the participants feel more comfortable.

In the present study TSI, tHb and O_2_Hb in the WHIIE dynamic recovery stage was significantly higher than that of LBEHIIE (Table 2). That means in the dynamic recovery stage, water-based exercise has higher tHb and muscle oxygen saturation than that on land ergometer exercise. Dynamic recovery is the buffer after high-intensity exercise. If more supplemental oxygen and tHb are available at this stage, the quadriceps will have a greater amount of oxygen to meet the next stage in the challenge. The possible reasons that explain the high TSI, O_2_Hb and tHb in WHIIE are the increase in hydrostatic pressure and buoyancy due to water environment movement. The heart blood volume during diastole in water is greater than that on land, therefore the stroke volume is increased, and the blood flow to the main action muscle group is increased^32,33^. Recent studies showed that more muscle oxygen utilization and muscle blood flow occur in water-based exercise than with land-based exercise, whether aerobic exercise, muscle exercise or aerobic combined strength courses^38,39^. The implication is that the oxygen and energy supplies are sufficient during muscle contraction in water, which can also accelerate energy metabolites elimination. In addition, the tHb in the main exercise stage presented a lower trend than the resting and dynamic recovery stages, whether in the water or on land ergometer. These results were consistent with the results of Tew et al.^40^ with the tHb decreased rapidly during exercise and increased rapidly over recovery and exceeded the resting values.

The blood lactate concentration is the net lactate production and elimination value. The blood lactate level is positively correlated with post-exercise fatigue^29,30^. In this study there was no difference in the lactate concentration between the two environments at exercise post immediately and in the 10th minute after exercise. However, there was a trend that WHIIE was lower than LBEHIIE at immediately and 10 min post-exercise (*p* = 0.062; *p* = 0.062). A previous study showed that participants performed 15 min of exercise at a pace of 1.15 Hz and 2.3 Hz to compare the difference in blood lactate concentration between land, shallow water, and deep water. The results showed that the blood lactate concentration in water was significantly lower than that on land^41^. Our previous study findings are also in line with the previous findings that the lactate concentration in 15 min of moderate intensity dynamic recovery in water following 6 min above moderate exercise intensity treadmill was lower than the dynamic recovery from similar exercise intensity on land^31^. The lower blood lactate concentration in water may be due to trunk blood redistribution caused by water hydrostatic pressure, which might, at least in part, lead lactate to be metabolized and cleared in other non-exercise muscles. Moreover, the vasodilation increased the amount of blood in the atrium, and the stroke volume increased after exercise, which accelerated the water exercise metabolites elimination speed^32^.

We found that the percentage of change in TSI during the dynamic recovery period was significantly negatively correlated with the change rate in lactate concentration at post-exercise in WHIIE (r = − 0.664). That reveals the more TSI increases during the dynamic recovery period, the less the lactate increase rate at post-exercise in WHIIE. The recent systematic review analyzed the effects of high levels of oxygen on post-exercise recovery in the last 15 years. It has been found that when exercise is performed at higher oxygen levels, higher muscle work and lower heart rate and RPE occur during exercise^42^. The present study showed that the WHIIE blood flow during the dynamic recovery period was higher than that of LBEHIIE [18.5 μM (12.1, 26.5) vs. 9.7 μM (5.9, 19.5)], and can be explained that it is possible to increase the blood lactate elimination rate due to higher blood flow, so that the change in blood lactate trend at the 10th minute after WHIIE is lower than LBEHIIE [146.7 (84.2, 232.0) vs. 277.8 (168.5, 463.7) %]. That means the lactate concentration at the 10th minute after WHIIE was closer to pre-exercise, and the lactate concentration declines faster. To the best of our knowledge, this study was the first to demonstrate a high rate of lactate concentration decline after HIIE in water, and the high rate of lactate concentration decline is associated with higher TSI, tHb and O_2_Hb in muscle tissue during the dynamic recovery stage.

### Limitation

As well known, subcutaneous adipose tissue thickness (SATT) influences NIRS data^43^. Unfortunately, in the present study, several participant’s quadriceps muscles SATT was too thick to collect correct data. We found the adipose tissue layer was more than 27 mm making it impossible for NIRS to detect and thus unable to collect data. This is consistent with previously study indicated SATT was greater than ~ 20 mm which interfered with the skeletal muscle measurement^44^. As a result, data were collected from only 11 participants in this study and the sample size is small. This is one of limitation of the present study. Several studies showed the maximal testing ability in water and on land^45,46^. However, we considered the participant’s age was 56 years old (53.0, 58.0) that was not appropriate to performed maximal testing. Therefore, we used the predicted maximum heart rate formula by age in the present study. This is the second limitation in the present study. At last, we chose the land-based bike ergometer which is mainly low impact and easy to obtain equipment. However, different models and different environmental factors may not be able to clearly clarify which factors lead to research results. This is the third research limitation of this study. A comparative water jumping exercise and bike ergometer study would be investigated in the future.

High-intensity intermittent exercise is an exercise mode that combines time efficiency, diversity, and fun^47^. Due to the decline in muscle power and muscle strength after middle-age^16^, the difficulty in performing HIIE on land is relatively high. The low-impact exercise pattern presents less joint pressure load on the lower limbs, HIIE in water and on land bike ergometer, are suitable for post-menopausal women. The present study found that WHIIE can achieve high cardiovascular exercise stimulation like LBEHIIE. However, there is less fatigue, faster heart rate recovery, and less WHIIE lactate elimination rate. In addition, the present study was the first study to confirm that the greater the TSI percentage change, the more the decline in lactate concentration during the dynamic recovery period in water.

In summary, this study demonstrated that HIIE in water produces less fatigue, higher lactate concentration decline rates, and faster heart beat recovery than HIIE on land bike exercise when achieving similar exercise intensity. WHIIE had a higher TSI, tHb and O_2_Hb in the dynamic recovery period than LBEHIIE. In addition, the greater the increase in TSI during the dynamic recovery period, the greater the decrease in lactate concentration. The findings from this study show that WHIIE is a suitable training mode for post-menopausal women to enhance endurance fitness.

## Methods

### Participants

We recruited 11 women aged 45–70 years old from the Taipei metropolitan area. These recruits had been in menopause for more than 1 year [age 56 years old (53.0, 58.0), BMI 20.0 kg/m^2^ (19.0, 22.4), and body fat rate 28.8% (26.0, 32.0)] and self‐reported that they were not regularly engaging in medium—to high‐intensity exercise (intensity > 6 METs). The exclusion criteria included hospitalization record within 3 months, symptomatic cardiovascular disease, uncontrollable hypertension or metabolic disease, inconvenience in movement caused by skeletomuscular injuries, neuromuscular or respiratory diseases, fear of water, skin diseases and cognitive disorders. Participants were advised not to engage in strenuous exercise for 3 days before the experiment and maintain their daily routine. The purpose and methods used in this study were described to all participants in detail before the experiment. All participants had sufficient and clear understanding of the purpose, experimental procedures, and possible involved risks. Informed consent was obtained from all study participants. This study was approved by the office of Human Research, Taipei Medical University (N201602073). This study conforms to Declaration of Helsinki for study involving humans.

### Experimental design and procedures

A randomized controlled crossover design was used in this study. Each participant visited the experimental site four times on different days. At the first visit to the experimental site the participants completed the health history, body composition measurement, land ergometer exercise intensity confirmation, and water-based movement practice. Instructor made sure the movement was correct before formal testing. When the participants visited the second time, four jump movements (counter-movement jump, CMJ; jumping jacks, JJ; single-leg jump, SLJ and lunge jump, LJ) intensity were determined by confirming the frequency of movements. We used a randomized counterbalanced sequence to determine the order of WHIIE or LBEHIIE on the third- and fourth visits. The two tests were separated by at least 7 days. Tests must be completed within 14 days to reduce the time interference effect.

**Figure 2:**
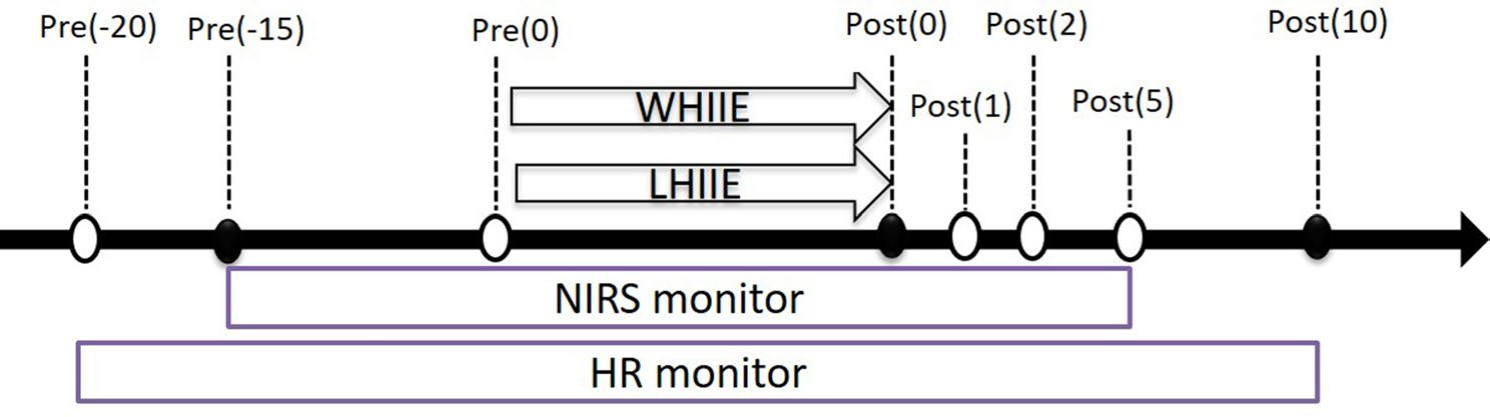
The proceeding measurement. ○ *The HR and RPE measurements;* ● the HR, RPE time and lactate concentration measurements; *WHIIE*, water high intensity interval exercise; *LBEHIIE*, land bike high intensity interval exercise; *Pre*, pre-testing; *Post*, post-testing.

The water depth in the water-based exercise test was hip level (90 cm). The water temperature was 32.2 ± 0.42 °C, and the humidity was 63.2 ± 2.40%. Participants wore heart rate monitors (Forerunner 920XT GPS; Garmin, Taiwan) and NIRS device (PortaMon; Artinis Medical System BV, Netherlands) after the ear lobe blood sampling. They stood in water for 5 min then measured the HR, muscle oxygenation parameters and record RPE in pre-exercise. A fitness instructor led the participants in performing warm‐up exercises in the water. Warm-up activities included 8 min of exercises (multi‐directional running at a comfortable speed for 2 min, movement re-familiarization for 3 min and stretching for 3 min (quadriceps, posterior leg muscles, calf muscles, iliopsoas, medial thigh muscles). The instructor cued participants to adjust their movements to achieve 80% heart rate reserved (HRR) ± 5 bpm (beat per minute) as high intensity and 50% of HRR ± 5 bpm as dynamic recovery intensity^48^. Each jumping set was performed at 120‐seconds interval as follows: 30 s of 80% HRR with 90 s of 50% HRR. There were eight sets in the exercise. All jumps were performed with the hands on the hips. Heart rate and RPE monitoring was measured and recorded during exercise (including main exercise and dynamic recovery), immediately, 1 min, 2 min, and 10 min post- exercise. NIRS (O_2_Hb, HHb, TSI, and tHb) performed at pre-exercise, during exercise, post-exercise and 5 min after exercise. Ear lobe blood sampling was performed at pre-exercise, immediately and 10 min post-exercise (Fig. 2). The maximum heart rate on land was calculated using the formula: 206.9 − (0.67 × age)^49^. The target heart rate was calculated using the formula: (Heart rate maximum − Heart rate resting) × (50 and 80)% + Heart rate resting. The maximum heart rate in water was calculated at 206.9 − (0.67 × age) − (land standing Heart rate − water standing posture Heart rate)^50^. LBEHIIE is bike ergonomic exercise testing on land. The temperature was 28.5 ± 1.58 °C and the humidity was 64.3 ± 2.33%. The speed was maintained at 70–80 rpm/min, with 30 s at 80% HRR followed with dynamic recovery intensity at 50% HRR for 90 s and repeated for eight sets. All parameters were performed at the same time as the WHIIT.

### Measurement

#### Water-based exercise test

##### Movement frequency confirmation

Participants wore heart rate monitors (Forerunner 920XT GPS; Garmin, Taiwan) before testing. They warmed up for 8 min before testing. The testing sequence of movement was CMJ, JJ, SLJ, and LJ. Instructor asked the participant to do jumping speed following cadence with audible metronome (model sQ100-88; Seiko Holdings Corp, Tokyo, Japan). The speed was increased by 5 beats every 25 s. Instructor observed the heart rate response during the process. When the heart rate reached the estimated 80% HRR, the jump movement lasted for 25 s then stopped. After 4 min of rest, the 80% HRR intensity and the movement speed of the next movement were confirmed. A total of four jump movements were confirmed in turn.

##### High-intensity interval test

Participants took warm up for 8 min. Each jumping set was performed as a 120‐second interval as follows: 30 s of 80% HRR with a previously confirmed movement speed, followed by a 90-s dynamic rest at 50% HRR exercise intensity. Two sets were performed for each movement. All jumps were performed with the hands on the hips with the lower body movements as follows: CMJ, JJ, SLJ, and LJ. A total of eight sets were performed in the exercise test.

#### Land ergometer exercise test

##### Exercise intensity confirmation

A land-based test was carried out using a stationary bicycle (Daum ergometer ergo; bike Premium 8i; Europe). The warm-up exercise on the ground included 2 min of multiple-dimension comfortable speed running, 3 min of muscle stretching (quadriceps, posterior leg muscles, calf muscles, iliopsoas, medial thigh muscles) and a 3-min ergometer to warm up (starting resistance 40 watts, speed 70–80 rpm). The exercise load was increased by increasing the resistance by 10 W/min in progression. The HR and RPE values per minute were recorded. When the heart rate reached the estimated 80% HRR, the speed and resistance remain unchanged for 1 min. The test was then terminated. We recorded the workload and HR time point at the estimated 40%, 50%, and 80% HRR. Previous studies showed that high intensity was 130% of the intensity associated with V̇O_2_max^51,52^. We used the extrapolation method to calculate the workload reaching 120% HRR according the pilot testing result. After 30 min of rest the participants were tested with a workload of 120% HRR for 30 s to confirm whether they reached the 80% HRR exercise intensity target.

##### High-intensity interval test

Participants took 8 min to warm up. The LBEHIIE test was subsequently performed. The testing intensity, time, and the number of sets of high intensity and dynamic rest were the same as the WHIIE testing.

#### Body composition

Body composition was measured using the Inbody 230 (Biospace Company, Ltd., Seoul, Korea). Participants did not exercise for 2 h before the measurement and were fasted for more than 2 h. Participants were asked to wear light clothing, take off their shoes and socks, and measure the contact between the hands and one foot with full contact with the electrode. The body composition ICC was from 0.96 to 1^53^.

#### Rating of perceived exertion

Borg’s scale was used as the exercise intensity assessment^54^. The value is 6–20 points. The smaller the values, the easier it feels. The greater the values, the more intense/tired it feels. Before the exercise testing, participants familiarized the meaning of scale. The RPE scores were recorded at 5 min pre-exercise, during exercise, dynamic recovery, immediately, 1, 2, and 10 min post-exercise.

#### Lactate

Approximately 20 μl of blood was collected from the participant’s ear lobe using a blood collection needle. The blood then flowed into the groove in the test piece (Lactate Scout+; EKF Diagnostics; Germany) to display the value. The measurement times were 5 min pre-exercise, immediately, and 10 min post-exercise. The exercise change rates were calculated immediately after exercise. The change rate formula was as follows:

$$\begin{aligned} {\text{Change rate immediately after exercise}} & = {{\left( {{\text{immediately after exercise}} - {\text{before exercise}}} \right)} \mathord{\left/ {\vphantom {{\left( {{\text{immediately after exercise}} - {\text{before exercise}}} \right)} {{\text{before}}}}} \right. \kern-\nulldelimiterspace} {{\text{before}}}} \\ & {\text{exercise}}*100\% \\ \end{aligned}$$

$$\begin{aligned} {\text{Change rate }}10\;\min \;{\text{after exercise}} & = \left( {10\;\min \;{\text{after exercise}} - {\text{before exercise}}} \right)/{\text{before}} \\ & {\text{exercise}}*100\% \\ \end{aligned}$$

#### Muscle oxygen saturation concentration measurement

**Figure 3:**
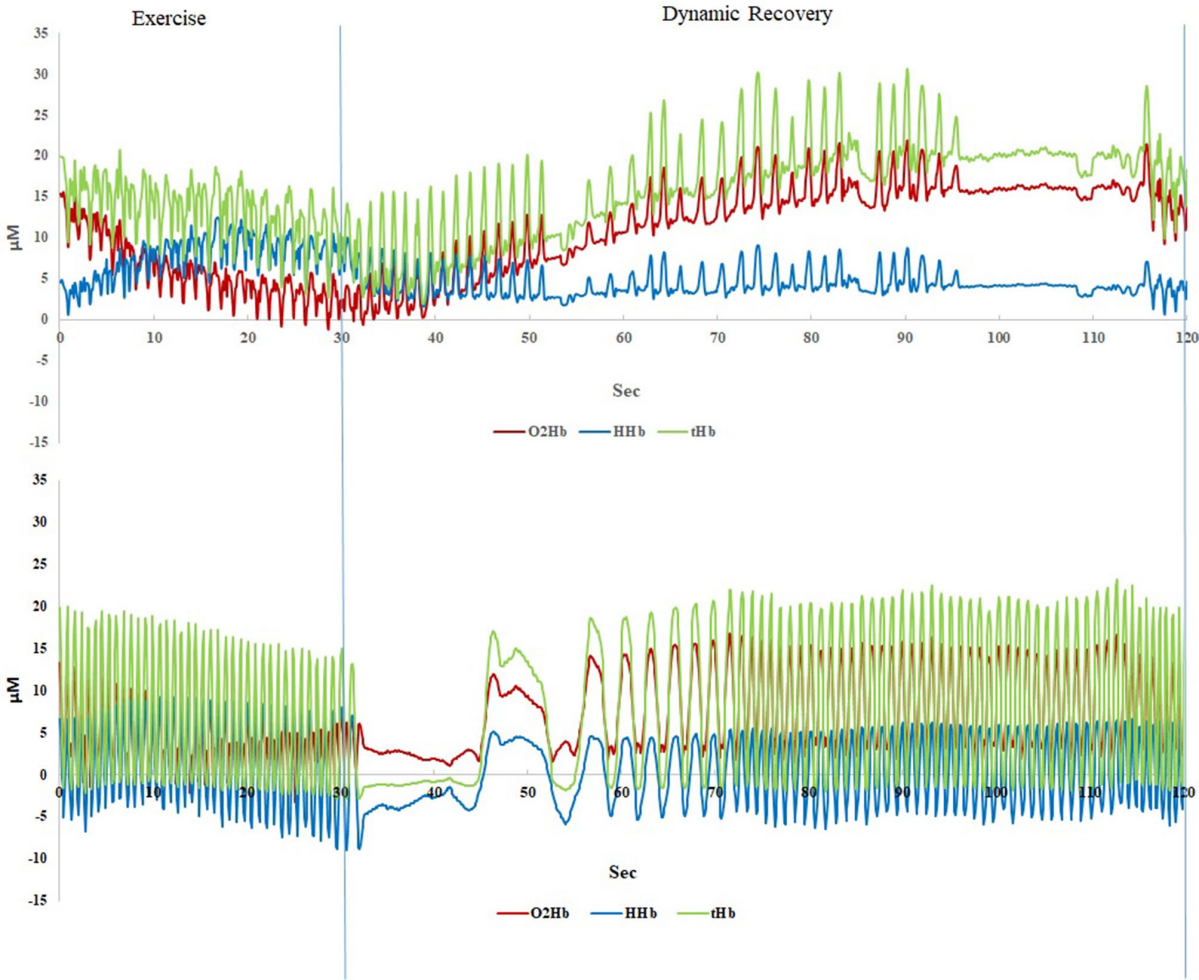
A representative participant’s quadriceps muscle oxygenation response in water-based and on land ergometer intermittent exercise test (1 set of main exercise and dynamic recovery). (**a**) Water high-intensity interval exercise. (**b**) Land high-intensity interval exercise. *O*_*2*_*Hb*, oxy-hemoglobin; *HHb*, deoxy-hemoglobin; *tHb*, total hemoglobin.

A portable and continuous-wave near-infrared spectroscopy (NIRS) (PortaMon; Artinis Medical System BV, Netherlands) was fixed in a vacuum sealed state using a film bag and a vacuum sealer. The NIRS device was placed on the rectus femoris of the right leg, 4 cm away from the patella superior border using 3 M double-sided tape and Hypatia. Make sure that device position does not move during exercise and does not impede the subject's movements. The device recorded data from 5 min pre-exercise, whole exercise testing process continuously, and end the recording 5 min after the exercise. The NIRS device consists of a light source with three wavelengths (λ = 760–850 nm). Three distances are used between receiver and transmitters: 30, 35 and 40 mm. We recorded the muscle tissue O2Hb, HHb, TSI and tHb during the jumping test. The sampling frequency was 10 Hz. The data analysis method calculated the O_2_Hb, HHb, TSI and tHb average value during the rest period (the last 20 s of 60 s), the high-intensity exercise (the last 20 s in each group of 30 s), the dynamic recovery stage (the last 20 s in each group of 90 s), and post-exercise for 5 min (the average of the last 20 s in each group at 1, 2, an 5th minutes (Fig. 3).

### Statistical analysis

Data processing and statistical analysis were performed using SPSS18.0 statistical software. All values were presented as median (IC rang). We compared TSI, tHb, O_2_Hb, HHb, heart rate, RPE and lactate concentration at various time points between WHIIE and LBEHIIE using Wilcoxon signed rank test. The correlation between the change percentage in TSI and lactate concentration in water and on land was determined using the Spearman correlation. A *P*-value of < 0.05 indicated a statistically significant difference. After acquiring test data from the eight participants, analysis was performed using G Power software (Heinrich‐Heine‐Universität Düsseldorf, Germany) with the power set 0.8. The TSI and lactate results were 0.73 and 071. The targeted sample size was 14. We recruited 17 postmenopausal women as participants. However, six participants had more than 27 mm adipose skinfold. Excessively thick adipose tissue disturbs the NIRS measurement^44^. A total of 11 participants completed the study. We also calculated the effect size and data power from 11 participants. The effect size from 0.53 to 0.63 (average 0.57) and the power was from 0.76 to 0.87 (average 0.81) with TSI, tHb and O_2_Hb. The effect size was 0.40 and the power of lactate data was 0.55 in immediately and 10 min post-exercise.
